# Physical Therapy Exercises for Sleep Disorders in a Rehabilitation Setting for Neurological Patients: A Systematic Review and Meta-Analysis

**DOI:** 10.3390/brainsci11091176

**Published:** 2021-09-05

**Authors:** Marco Tramontano, Sara De Angelis, Giovanni Galeoto, Maria Carmela Cucinotta, Danilo Lisi, Riccardo Maria Botta, Mariagrazia D’ippolito, Giovanni Morone, Maria Gabriella Buzzi

**Affiliations:** 1 Fondazione Santa Lucia IRCCS, 00179 Rome, Italy; s.deangelis@hsantalucia.it (S.D.A.); maracucinotta3@gmail.com (M.C.C.); mg.dippolito@hsantalucia.it (M.D.); g.morone@hsantalucia.it (G.M.); mg.buzzi@hsantalucia.it (M.G.B.); 2 Department of Human Neurosciences, Sapienza University of Rome, 00185 Rome, Italy; giovanni.galeoto@uniroma1.it; 3 IRCCCS Neuromed, 86077 Pozzilli, IS, Italy; 4 Azienda Ospedaliera Rilievo Nazionale Sant’Anna e San Sebastiano, UOC Risk Management, 81100 Caserta, Italy; danilolisi@libero.it; 5 Azienda Ospedaliera Universitaria Mater Domini, 88100 Catanzaro, Italy; bottariccardimaria@gmail.com

**Keywords:** neurological diseases, sleep disorders, physical therapy, neurorehabilitation

## Abstract

Background: Sleep occupies one-third of human life and is essential for health and for emotional, physical, and cognitive well-being. Poor or insufficient sleep is associated with a wide range of dysfunctions that involve different body systems, such as the endocrine, metabolic, and immune systems, thus compromising the higher cortical functions, cognitive performance, mood, and post-physical activity recovery. The present systematic review and meta-analysis aimed to explore the effectiveness of physical therapy exercises on sleep disorders in patients with neurological disorders. Our systematic review identified 10 articles that investigated the effects of physical therapy on sleep disorders in patients with neurological disorders, 6 of which were included in the meta-analysis. Results suggest that physical therapy exercises are a safe and useful strategy for managing sleep disorders in neurorehabilitation.

## 1. Introduction

Sleep is a common function of living species. It occupies one-third of human life, and it is shown to be essential for health and for emotional, physical, and cognitive well-being [1,2,3,4]. Poor or insufficient sleep is associated with a wide range of dysfunctions that involve different body systems, such as the endocrine, metabolic, and immune systems, thus compromising the higher cortical functions, cognitive performance, mood, and post-physical activity recovery [1,4]. Sleep disturbance can affect both the duration and the quality of sleep, and when it occurs, it reduces the functionality and quality of life (QoL) of the person. Additionally, it represents a risk factor for secondary diseases and medical conditions [5]. Sleep quantity and quality can be affected by age, physical and psychological conditions, and environmental factors [4]. Several studies have shown that sleep disorders (insufficient sleep, excessive amount of perceived sleep, abnormal movements during sleep) are common among the non-motor symptoms in patients with neurological disorders [1,6,7,8]. 

Excessive daytime sleepiness, likely caused by a combination of alterations in pathophysiological mechanisms involved in the regulation of sleep/wakefulness, effects of dopaminergic drugs, and nocturnal sleep disruption, is common in patients with Parkinson’s disease (PD) [7,8,9,10,11]. Likewise, in patients with multiple sclerosis (MS), daytime sleepiness represents a disturbance that contributes to fatigue and other chronic MS symptoms, highly impacting the patient’s activities of daily living (ADL) [12,13]. 

Sleep disorders are frequently identified following traumatic brain injury (TBI); poor sleep efficiency, short sleep duration, long sleep onset, hypersomnia, and sleep-related breathing disorders have been reported [14,15]. Sleep apnea, insomnia, restless legs syndrome, and daytime sleepiness are common in stroke survivors, affecting not only the ADL and QoL of both patients and caregivers but also representing a high risk of further cerebrovascular events [6,16,17].

Moreover, studies have shown that the quality and quantity components of sleep can be compromised by the condition of hospitalization [18,19].

During neurorehabilitation, sleep disorders can potentially affect the recovery process, through reduced ability to engage in physical therapy activities [20,21,22,23,24].

In addition, there is evidence that sleep disorders and depression might lead to cognitive problems, in particular related to the learning process, with limited benefits from the use of sedatives (i.e., reduced memory and learning consolidation and recall after stroke) [25,26]. Impaired motor learning might also affect rehabilitation outcomes during the neurorehabilitation process as a learning mechanism fundamental to harnessing neuroplasticity [27].

Thus, it may be helpful for patients with neurological diseases to carry out an assessment of the overall sleep pattern and eventually to provide appropriate management and better rehabilitation outcomes [28,29]

Although pharmacological treatment is widely used in the general population with sleep disorders, medication use in neurological patients who are undergoing rehabilitation may be problematic, because of possible adverse effects due to some sedative-hypnotic drugs [30,31]

Among non-pharmacological treatments, physical exercise has been suggested as an activity that may improve sleep disorders [32]. Studies in healthy participants have shown that physical exercise may improve both the quality and the quantity of sleep [32,33,34]. Furthermore, physical therapy may be considered a powerful non-pharmacological intervention for sleep disorders also in neurological disease, with consequent positive effects on both motor and non-motor functions and with minimal side effects [31,35,36,37,38]. Although physical therapy exercises may improve sleep disorders in neurological patients, they are often neglected in conventional neurorehabilitation programs.

In light of the above findings, this systematic review and meta-analysis aims to analyze the effects of physical therapy on sleep disturbances, performed in a clinical setting, in patients with neurological disorders in order to identify specific protocols that could be included in individualized conventional neurorehabilitation programs in different clinical settings.

## 2. Materials and Methods

This systematic review was performed in accordance with Preferred Reporting Items for Systematic Reviews and Meta-Analyses (PRISMA) statement [39] and the Cochrane Handbook for Systematic Reviews of Interventions [40]. The study’s protocol was registered on the website of the PROSPERO International prospective register of systematic reviews (registration number CRD42021250760).

### 2.1. Search Strategy and Eligibility Criteria

Electronic databases searched in April 2021 were MEDLINE (PubMed) and Physiotherapy Evidence Database (PEDro). The search terms used were (sleep disorder*) AND (rehabilitation) AND (physical therapy modalities). The search terms were modified for each database, and appropriate subheadings were used for each database searched (for details, see Appendix A). The combination of search terms was defined using the population, intervention, comparison, and outcome (PICO) model. The population was limited to neurological patients; interventions included all the supervised physical rehabilitative protocols, manual therapy, and robotic rehabilitation; the comparison was evaluated considering no intervention, unsupervised home-based exercises, standard medical care, and other types of therapies/protocols different from the supervised physical one; and outcomes included any changes shown by the patients in sleep disorders, assessed instrumentally and/or clinically. Both studies that considered changes in sleep disorders as a primary outcome and studies that investigated changes in sleep quality and quantity as a secondary outcome were included in the present review. 

Controlled and non-controlled clinical trials (i.e., randomized and non-randomized trials), retrospective studies, case reports, case series, and observational studies were included. No restrictions related to publication date, sex, and country were applied. Studies with a published full text in English or Italian were considered eligible. 

Reviews, studies based on pharmacological treatments, and studies that included patients without neurological diseases were excluded. 

### 2.2. Study Selection and Data Collection Process

Duplicate records were identified and removed using EndNOTE software. Study eligibility assessment and the data extraction process were carried out by two independent co-authors (SDA and MCC). In the case of any disagreement, the opinion of a third author (MT) was used to reach an agreement. The first selection of studies was initially conducted considering the title and abstract; afterward, full-text articles were examined. 

The summary of results was reported following the Preferred Reporting Items for Systematic Reviews and Meta-Analyses (PRISMA) statement [39]. Two authors (DL and RMB) independently extracted the following relevant features of the included studies: name of the first author and publication year, study type, participants, rehabilitative intervention, and outcome measures. 

### 2.3. Risk of Bias

Following the instructions in the Cochrane Handbook for Systematic Reviews of Interventions, the risk of bias was assessed using six criteria that were individually rated for each study. In this context, selection bias, performance bias, detection bias, and attrition and reporting bias were considered by the reviewer and assessed using the PEDro score.

The risk of bias was assessed using the Cochrane risk of bias [41] for the controlled trials and using a modified version of the Newcastle–Ottawa Scale (NOS) [42] for the observational studies. The assessment was performed by two authors (SDA and MCC); discrepancies were resolved by consensus with a third reviewer (MT). The modified NOS ranges from 0 to 7. In both scales, the higher is the score, the better is the methodological quality. 

### 2.4. Data Synthesis

Data concerning qualitative synthesis were reported in a descriptive way by using means, DS, percentages, and ranges. 

Quantitative analysis was conducted by comparing the outcomes used in the included studies. The studies’ follow-up results were also considered and pooled.

When available, data for continuous variables were reported as mean differences (MDs), along with their 95% confidence intervals (CIs), while dichotomous outcomes were reported as relative risks (RRs), along with their 95% CIs. In the case of missing data, the authors of the studies were contacted for further information.

A meta-analysis of either dichotomous outcomes or continuous outcomes was carried out whenever possible; when a meta-analysis was not possible, results were presented using summary and descriptive statistics. Meta-analyses were carried out using Review Manager (version 5.2.6, Cochrane Collaboration, Oxford, England), and the *p*-value was considered statistically significant at <0.05. 

## 3. Results

**Figure 1:**
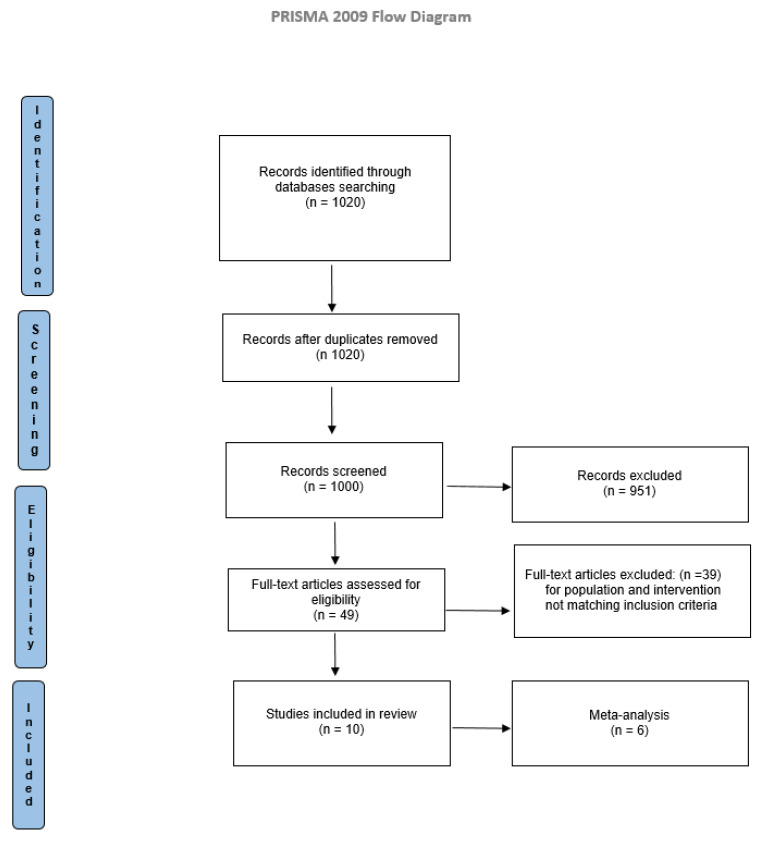
PRISMA flow diagram.

Electronic searches identified 1020 studies. Titles and abstracts were examined according to eligibility criteria. The full texts of the articles were read to determine the eligibility. Comparison of the retrieved titles identified 20 duplicates, which were excluded. The result consisted of 1000 articles eligible for inclusion. After a full-text analysis, 990 did not match the inclusion criteria, and 10 studies were included in the present systematic review, as reported in Figure 1.

Table 1 presents a narrative summary of results including studies with their associated characteristics and patient features. In particular, the following data are reported: first author’s name, publication year, study type, participants, intervention, intervention duration, and outcome measures. 

The included studies were all published in English and were conducted in different countries: three studies came from the United States, three came from Brazil, and Japan, Iran, Jordan, and Switzerland contributed to this review with one study each. Of the five investigated neurological diseases, four studies included patients with a diagnosis of PD, three studies included patients with stroke, two studies involved patients with a diagnosis of multiple sclerosis (MS), and one study included patients with spinal cord injury (SCI) between T7 and T12; no studies regarding patients with TBI were found.

In addition, 747 patients with neurological diseases and sleep disturbance were included in the review, of whom 490 had a stroke diagnosis, 133 had a clinical diagnosis of PD, 111 were persons with MS, and 13 were patients with SCI between T7 and T12. 

Among the 10 included studies, 6 studies were randomized controlled trials (RCTs) [43,44,45,46,47,48] and the other 4 studies were a non-randomized controlled trial [49], a non-controlled trial [50], an exploratory pre–post-test investigation [51], and a one-group pre-test and post-test [52].

**Figure 2:**
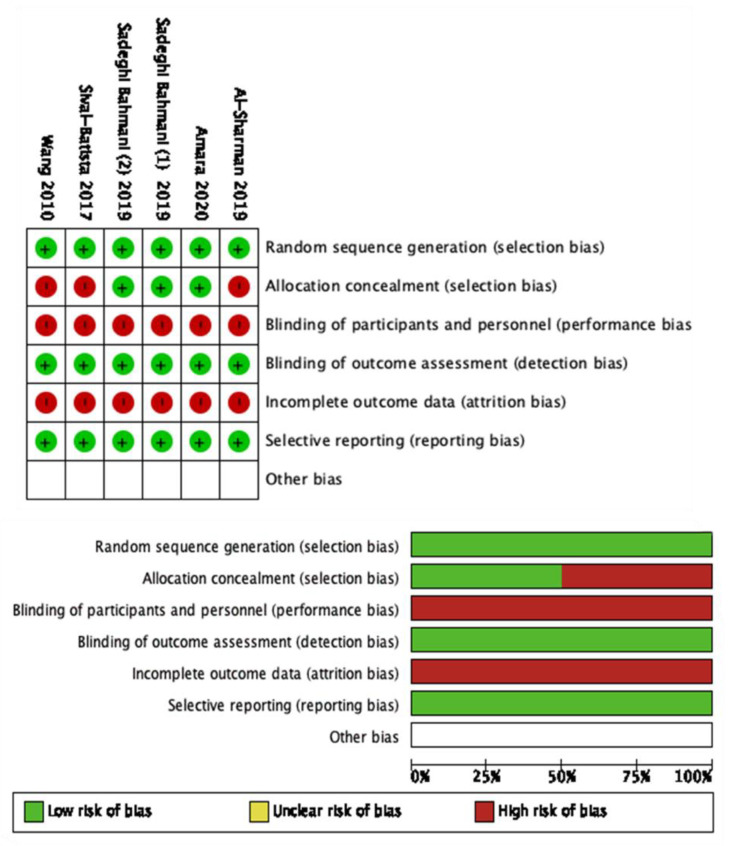
Cochrane risk of bias of the included studies.

The modified NOS scale was used to assess the quality of non-RCTs. The NOS scale of the included studies ranged between 5 to 6, with a mean score of 5.4 points out of 7 (Table 2). None of the included studies reached the maximum score. The Cochrane risk of bias [41] was used for the RCTs (Figure 2). 

The primary aim of the included studies was to evaluate the effect of a physical therapy intervention on sleep disorders in patients with neurological diseases. 

All the included studies carried out a supervised physical therapy intervention. Different types of protocols in terms of proposed exercises and duration were performed. Summarizing the data, the physical therapy interventions lasted between 6 weeks and 3 months and with one to five sessions per week.

Concerning the outcomes, either instrumental or clinical assessments were performed to evaluate the sleep quality and quantity. The instrumental assessments consisted of polysomnography, electroencephalogram (EEG), electromyogram (EMG), electrooculogram (EOG), and actigraphy. Clinical scales, tests, and questionnaires were used to clinically assess sleep disorders and to investigate the patients’ self-assessment of sleep quality. All the outcomes are displayed in Table 1.

### 3.1. Meta-Analysis

Quantitative analysis was carried out by comparing outcomes and follow-ups. This pool was based on comparable outcomes, and comparable times of follow-up allowed consideration of six studies in the meta-analysis (Figure 2). A description of the experimental protocol and control therapy is given in Table 1.

These six studies are as follows.

#### 3.1.1. *Comparison Assessed with the Pittsburgh Sleep Quality Index (PSQI)*

The studies by Al-Sharman et al. [45], Amara et al. [44], Silvia- Batista et al. [47], and Wang et al. [48] were considered. Meta-analysis revealed statistically significant results (*p* < 0.00001) in favor of the experimental group compared to the control group (mean difference = −2.57, 95% confidence interval (CI) = −3.31, −1.81) (Figure 2). 

Al-Sharman et al. [45] compared the effects of a moderate-intensity aerobic exercise program (MAE) with a home exercise program (HEP) in individuals with MS. Both the interventions were conducted for 18 sessions, three times a week for 6 weeks, and each session lasted approximately 50–60 min + 15 min of stretching exercises before and after each exercise session. In Amara et al. [44], the effects of a resistance training (RT) intervention lasting three times a week for 16 weeks were compared with a sleep hygiene intervention (SHI) of a duration of 30–60 min + a telephone call every 4 weeks in patients with PD. Silvia-Batista et al. [47] compared an RT intervention of 24 sessions, two times per week for 3 months with a no-exercise intervention in PD. Wang et al. [48] compared the effect of a rehabilitation program based on physical exercises with a tai chi program in patients with stroke. Both the interventions were carried out once a week for 12 weeks. 

#### 3.1.2. *Comparison Assessed with the Fatigue Severity Scale (FSS)*

The studies by Amara et al. [44] and Sadeghi Bahmani et al. [46] were considered. Meta-analysis revealed statistically significant results (*p* = 0.06; mean difference = −3.97, 95% confidence interval (CI) = −8.18, −0.25) (Figure 2).

Amara et al. [44] compared the effects of the RT intervention lasting three times a week for 16 weeks, with an SHI of a duration of 30–60 min + a telephone call every 4 weeks in patients with PD. In Sadeghi Bahmani et al. [46], an endurance training (ET) intervention and a coordinative training (CT) intervention were compared with an active control condition (ACC) in patients with MS. The three interventions were carried out three times a week for about 45–60 min for 8 consecutive weeks.

#### 3.1.3. *Comparison Assessed with the Insomnia Severity Index (ISI)*

The studies by Amara et al. [44] and Sadeghi Bahmani et al. [46] were considered. Meta-analysis revealed statistically significant results (*p* = 0.06; mean difference = −1.78, 95% confidence interval (CI) = −3.61, −0.04) (Figure 2).

Amara et al. [44] compared the effects of the RT intervention lasting three times a week for 16 weeks with the SHI of a duration of 30–60 min + a telephone call every 4 weeks in patients with PD. In Sadeghi Bahmani et al. [46], the ET intervention and the CT intervention were compared with an ACC in patients with MS. The three interventions were carried out three times a week for about 45–60 min for 8 consecutive weeks

#### 3.1.4. *Comparison Assessed with Wake after Sleep Onset (WASO) Minutes*

The studies by Amara et al. [44] and Al-Sharman et al. [45] were considered. Meta-analysis revealed statistically significant results (*p* = 0.0002; mean difference = −46.70, 95% confidence interval (CI) = −70.85, −22.55) (Figure 2).

In Amara et al. [44], the effects of the RT intervention lasting three times a week for 16 weeks were compared with the SHI of a duration of 30–60 min + a telephone call every 4 weeks in patients with PD. Al-Sharman et al. [45] compared the effects of an MAE with an HEP in individuals with MS. Both the interventions were conducted for 18 sessions, three times a week for 6 weeks, and each session lasted approximately 50–60 min + 15 min of stretching exercises before and after each exercise session.

#### 3.1.5. *Comparison Assessed with Sleep Efficiency*

The studies by Amara et al. [44] and Al-Sharman et al. [45] were considered. Meta-analysis revealed statistically significant results (*p* = 0.0008; mean difference = 8.80, 95% confidence interval (CI) = 3.66, 13.94) (Figure 2).

Amara et al. [44] compared the effects of the RT intervention lasting three times a week for 16 weeks with the SHI of a duration of 30–60 min + a telephone call every 4 weeks in patients with PD. Al-Sharman et al. [45] compared the effects of an MAE with an HEP in individuals with MS. Both the interventions were conducted for 18 sessions, three times a week for 6 weeks, and each session lasted approximately 50–60 min + 15 min of stretching exercises before and after each exercise session.

#### 3.1.6. *Comparison Assessed with the Total Sleep Time (TST) Minutes*

The studies by Amara et al. [44] and Al-Sharman et al. [45] were considered. Meta-analysis revealed statistically significant results (*p* = 0.002; mean difference = 42.50, 95% confidence interval (CI) = 15.38–69.63).

Amara et al. [44] compared the effects of the RT intervention lasting three times a week for 16 weeks with the SHI of a duration of 30–60 min + a telephone call every 4 weeks in patients with PD. Al-Sharman et al. [45] compared the effects of an MAE with an HEP in individuals with MS. Both the interventions were conducted for 18 sessions, three times a week for 6 weeks, and each session lasted approximately 50–60 min + 15 min of stretching exercises before and after each exercise session.

**Figure 3:**
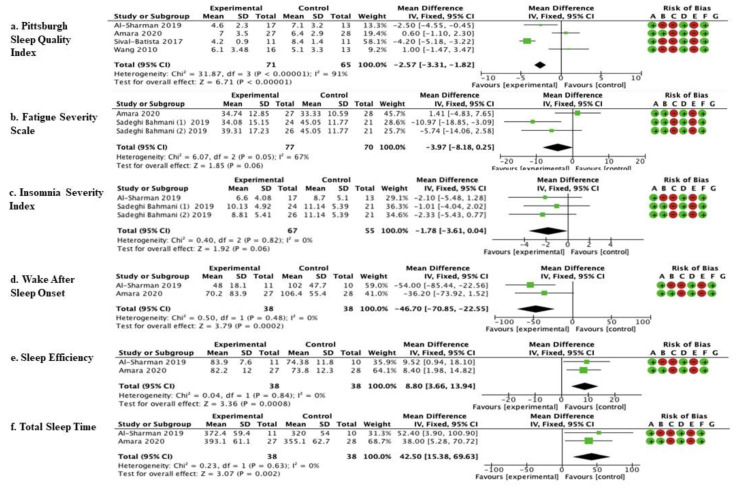
Comparison of physical therapy vs. comparison therapy assessed with clinical scales. 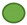 low risk of bias, 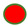 high risk of bias.

Figure 3 Show the results of the meta-analysis carried out on six studies by comparing different outcomes and follow-ups. This pool was based on comparable outcomes and comparable times of follow-up. The mean, standard deviation (SD), total number of participants, and data for continuous variables were reported as the mean difference, along with their 95% confidence intervals (CIs) for each study. The risk-of-bias summary for RCTs was reported for each of the six included randomized controlled trials. Scores from the Cochrane Assessment of Bias were reported for each clinical scale: a: PSQI; b: FSS; c: Insomnia; d: WASO; e: Sleep Efficiency; f: Total Sleep Time; experimental arm: supervised physical therapy; control arm: unsupervised exercises; no physical therapy; standard medical care; and other types of therapies/protocols different from the supervised physical one. 

## 4. Discussion

The present systematic review and meta-analysis was performed to analyze the role of physical therapy performed in a clinical setting in the improvement of sleep disturbances in neurological patients in order to identify specific protocols that could be included in individualized neurorehabilitation programs. 

Results suggest that physical therapy exercises could represent a beneficial interventional for improving sleep disorders in neurological patients. However, due to the relatively few studies and the heterogeneity of the interventions, it is difficult to generalize the results. Moreover, protocols differ in duration, intensity, and required tasks.

Most of the included studies explore the effects of physical therapy on sleep disorders in patients with PD [44,47,49,52], showing positive results. Specifically, multimodal physical therapy programs that stimulate aerobic metabolism and muscle endurance seem to be useful in improving sleep quality assessed by PSQI and the Mini-Sleep Questionnaire (MSQ), in addition to enhancing the sleep efficiency objectively measured with polysomnography [44,47,48]. Moreover, a decrease in the use of drugs promoting sleep was observed in patients following a physical therapy program, supporting physical exercises as a valid alternative in the non-pharmacological treatment of sleep disorders in patients with PD [44]. Interestingly, Amara et al. [44] highlighted how even an intervention based on sleep hygiene can lead to an improvement in the sleep quality assessed by the PSQI.

Although the PSQI is a subjective tool that can be conditioned by a placebo effect [53], the results shown by Amara et al. [44] leave open the possibility of linking a physical therapy program with discussion time with a board-certified sleep medicine physician to provide patients with suggestions for improving sleep hygiene. Further studies could investigate the effectiveness of this combined approach, and furthermore, they could identify the possible role of the physiotherapist in sleep hygiene programs.

Tidman et al. [52] showed an improved willingness to participate in social situations, perceived improvements in flexibility, and perceptions of improved daytime sleepiness in PD patients who performed a supervised physical exercise program, but no significant improvements were found in the sleep quality. These can lead to better adherence and responsiveness of the patient to the rehabilitation treatment, to an increased participation in ADL, and therefore to an improvement in the QoL [54]. 

Among the included studies, only one [45] evaluated sleep-related biomarkers (melatonin, serotonin, cortisol, highlighting that in patients with MS, aerobic training of moderate intensity leads to an increase in serotonin values. Moreover, this result, associated with the clinical and instrumental evaluation of the quality and quantity of sleep, shows a correlation between the increase in serotonin and the improvement in the quality of sleep assessed by the PSQI and the Insomnia Severity Index (ISI) after 6 weeks of physical training. 

These findings are also supported by the other included study concerning MS, which showed a decrease in subjective sleep complaints after 8 weeks of physical exercise programs [46]. Although the lack of further studies prevents us from defining the efficacy of treatment in patients with MS, these results suggest that 45-60 min of exercise sessions, three times a week for a total of 18-24 sessions, may lead to an improvement of insomnia in patients with MS.

Three studies explored the effect of physical therapy treatment on post-stroke patients with sleep disorders, without clinically significant results. Specifically, they compared the effects of conventional motor rehabilitation [48], robotic-based rehabilitation [43], a home exercise program [43], and specific and individualized supervised motor training [51]. The difference between the four rehabilitation protocols and the heterogeneity of the population (different types of lesions and different times from stroke onset) do not allow us to draw conclusions.

One study [50] observed the effect of an aerobic physical exercise protocol in patients with SCI between T7 and T12 associated with periodic leg-movement-related sleep disorders. Although results identified a significant improvement in sleep quality in these patients, the lack of other studies and the small number of patients (n = 13) do not allow conclusions, although the data can be considered as a promising observation for further studies. 

The heterogeneity of the studies makes it difficult to identify a single protocol that could be useful in improving sleep disorders in patients with neurological disorders. However, the presence of different protocols in terms of proposed exercises, settings, and populations could be considered as evidence of the great versatility and applicability of physical exercise in patients with neurological diseases associated with sleep disorders. Although physical therapy exercises for sleep disorders could be easily implemented in neurological patients, they are often neglected in conventional neurorehabilitation programs [55].

### Limitations of the Current Review

Several limitations in the present review and meta-analysis are acknowledged. First was the small number of studies for each investigated pathology. Second, the methodological heterogeneity (e.g., study designs, outcome measures) restricted the number of studies eligible for quantitative analysis. In addition, data reporting was frequently incomplete or not always provided in a useful way to perform meta-analysis. Third, the variability of the interventions did not allow us to identify a single rehabilitative protocol that verifies the effectiveness. The internal validity of studies was also limited, and the methodological quality was low to medium, on average, as a consequence of the study designs (lack of randomization and blinding, small or uncontrolled groups). Despite the fact that sleep has a great impact on rehabilitation outcomes, there is a lack of primary research that considers sleep disturbance as a primary outcome in neurorehabilitation. Thus, we considered studies that investigated changes in sleep quality and quantity as a secondary outcome, and this aspect might have limited our findings. 

## 5. Conclusions

Our review identified 10 articles that investigated the effects of physical therapy on sleep disorders in patients with neurological disorders, and 6 of them were included in a meta-analysis. Results suggest that physical therapy exercises could be a useful strategy for managing sleep disorders in neurorehabilitation. However, due to the heterogeneity of the interventions, it is difficult to generalize the results with a clinical recommendation. Future research in this area would benefit from a higher methodological quality of the study and interventions in physical therapy targeted specifically at sleep disorders. 

## Figures and Tables

**Table 1:** Included studies according to the PICO strategy.

Author, Year	Study Type	Population	Intervention	Comparison	Outcome Measures	Setting
Tidman, 2020 [52]	One-group pre-test and post-test	PD (n = 22) Hoehn and Yahr stage between 1 and 3 Mean age 72.95 ± 7.8 years	Community-based exercise program (rotation, flexibility, coordination, balance, and timing in response to demonstration by class instructors). Duration: 60 min a day, 3 times/week, for 8 weeks.	NA	- ESS	Tertiary center
Fulk, 2020 [43]	RCT	Stroke (n = 408) Group no sleep problem (NSP): 310 Sleep problems with no-to-minimal impact on function (SP-NMI): 64 Sleep problems with moderate or quiet impact on function (SP-MQBI): 34 Mean age 62.0 ± 12.7 years	NSP: locomotor training with body weight support (at 2 months post stroke). SP-MQBI: late locomotor training with body weight support (at 6 months post stroke). Duration: between 30 and 36 sessions, 3 times/week for 12 to 16 weeks, each session lasting 90 min.	SP-NMI: home exercise program (at 2 months post-stroke) Duration: between 30 and 36 sessions, 3 times/week for 12 to 16 weeks, each session lasting 90 min	- SIS - Self-reported impact of sleep problems on health-related quality of life (questionnaire)	Hospital
Amara, 2020 [44]	RCT	PD (n = 55) Resistance training (RT): 27 Sleep hygiene intervention (SHI): 28 Hoehn and Yahr stages between 2 and 3 Age ≥ 45 years	RT: combination of resistance training and body weight functional mobility exercises with limited rest intervals that we previously used in PD to challenge strength, power, balance, and endurance. After a familiarization session, resistance training volume and intensity progressed during a ramp-up phase over the first 4 sessions by increasing the number of sets (i.e., first day, 1 set; second day, 1 set; third day, 2 sets; and fourth day, 3 sets). Thereafter, RT intensity/training loads targeted 10 repetitions maximum (10 RM) in sessions 1 and 3 each week. For session 2, resistance loads were reduced by ~30%, with greater emphasis on maximizing the speed of movement during the concentric phase (eccentric phase was controlled/slowed) for 12 repetitions/set. Duration: 3 times/week for 16 weeks.	SHI: Participants randomized to the sleep hygiene intervention received suggestions for improving sleep hygiene through discussion with a board-certified sleep medicine physician. Duration: 30–60 min + telephone call every 4 weeks to address any questions about sleep hygiene measures	- PSG - PSQI - ESS - FSS - PVT	Tertiary center
Al-Sharman, 2019 [45]	RCT	MS (n = 40) Moderate-intensity aerobic exercise (MAE) program = 20 Home exercise program = 20 EDSS score of 3–5.5 PSQI > 5 Duration of disease: 9.6 ± 8.49 (MAE); 5.43 ± 4.2 (HEP) Mean age: 38.7 ± 13 years (MAE); 31.9 ± 10 years (HEP)	MAE: MAE + stretching exercises before and after each exercise session. Duration: 18 sessions, 3 times/week for 6 weeks. Each session lasted approximately 50–60 min + 15 min before and after each exercise session (stretching exercises).	HEP: home exercise program + stretching exercises before and after each exercise session Duration: 18 sessions, 3 times/week for 6 weeks, each session lasting approximately 50–60 min + 15 min before and after each exercise session (stretching exercises)	- PSQI - ISI - Actigraphy (Actigraph wGT3X-BT, Pensacola, FL, USA) - Biomarkers (melatonin, cortisol)	Hospital
Sadeghi Bahmani, 2019 [46]	RCT	MS (n = 71) Endurance training (ET): 24 Coordinative training (CT): 26 Active control condition (ACC): 21 Female; EDSS < 6; age range 18-65 years	ET: endurance training 25–35 min on a treadmill, exercise bicycle, or walking/jogging with individual pauses of 1–2 min, followed by 5 min of cooling down. CT: 30–45 min/session. 5 min of warming up, exercises focused on CT as balancing on a small bar, mirroring and imitating instructors’ movements, balancing balls, mirroring participants’ bouncing with balls of different sizes, surfaces, and weights, “football-tennis,” balancing with closed eyes on a rope on the floor, and similar exercises. Duration: 3 times/week for about 45–60 min for 8 consecutive weeks.	ACC: active control condition met 3 times/week for 30–45 min/session at the hospital center to ensure that frequency, duration, and the degree of social contacts of the control condition were identical to the endurance and resistance training conditions Duration: 3 times/week for about 45–60 min for 8 consecutive weeks	- ISI - BDI-FS - FSS	University hospital
Colledge, 2017 [51]	Exploratory pre–post-test investigation with a 6-month follow-up	Stroke (n = 48) Aneurysmal subarachnoid hemorrhage (aSAH) Mean age 58.5 ± 12.4 years	Individualized exercise program with a total energy expenditure of 17.5 kcal/kg/week for each participant (walking techniques, explained flexibility and motor skill learning tasks, and taught behavioral skills). Duration: 3–5 times/week over 12 weeks. Single exercise sessions lasted between 30 and 45 min.	NA	Night sleep EEG (Fp2-A1; electro-oculogram; electromyogram; SOMNOwatchTM, Randersacker, Germany)	University hospital
Silva-Batista, 2017 [47]	RCT	PD (n = 22) Resistance training (RT): 11 No exercises (NE): 11 Hoehn and Yahr stage between 2 and 3 Not presenting diagnosis for insomnia Age range 64–75 years	RT: 5 resistance exercises (leg-press, latissimus dorsi pull-down, ankle plantar flexion, chest-press, and half-squat), twice a week. Warm-up on a cycle ergometer (20–40 rpm). A linear periodization in which the training load progressed from high-volume, low-intensity to low-volume, high-intensity loads over 12 weeks was implemented in an attempt to maximize training adaptations. An interval of 2 min was allowed between exercises and sets. Duration: 24 sessions, 2 times/week for 3 months, each session lasting 50 min.	NE: no exercise training activities	- PSQI - Knee extensor peak torque (isokinetic dynamometer Biodex System 3; Biodex Medical Systems, Shirley, NY, USA)	Tertiary center
Nascimento, 2014 [49]	No randomized controlled trial	PD (n = 34) Multimodal exercise program (MEP): 17 Standard medical care (SMC): 17 Hoehn and Yahr stages 1 through 3 Mean age 67.05 years	G1: multimodal exercise program to stimulate aerobic metabolism: warm-up, muscular resistance, balance and motor coordination, and aerobic fitness. Duration: 1 session/week for 6 months, each session lasting 60 min	SMC: standard medical care routine	- PIAQ - MSQ	University clinical center
Wang, 2010 [48]	RCT	Stroke (n = 34) Tai chi (TC): 17 Non-resistance training (NRT): 17 Intracerebral hemorrhage, subarachnoid hemorrhage, or cerebral infarction Age ≥ 50 years	TC: tai chi program based on classical Yang style (warm-up, practice, cool down). Duration: once a week for 12 weeks, each session lasting 50 min.	NRT: rehabilitation program Non-resistance training (such as walking and/or standing and resistance training using exercise machines and Thera-Band Tubing Duration: once a week for 12 weeks, each session lasting 80 min	- 60-item GHQ - PSQI	Hospital
De Mello, 2002 [50]	No controlled trial	SCI (n = 13) Males; spinal cord injury between T7 and T12 and total injury to the upper motoneurons Clinically stable Mean age 31.6 ±8.3 years	Aerobic physical exercise: The protocol of the maximum effort test consisted of a 2 min warm-up with a load of 25 watts in 5 watts/min load increments until exhaustion, with 3 min of active recovery at a load of 25 watts. The mean rotation speed was 70 ± 80 rpm. Duration: 45 days of consecutive sessions, 3 times/week, each session lasting a mean of 30 min.		- PSG (Oxford/Medilog 8 channels) (3 EEG channels, 2 EOG channels, and 3 EMG channels)	Clinical center

BDI-FS = Beck Depression Inventory-Fast Screen; EDSS = Expanded Disability Status Scale; EEG = electroencephalogram; EMG = electromyogram; EOG = electrooculogram; ESS = Epworth Sleepiness Scale; FSS = Fatigue Severity Scale; G = group of intervention; GHQ = general health questionnaire; ISI = Insomnia Severity Index; MS = multiple sclerosis; MSQ = Mini-Sleep Questionnaire; PD = Parkinson’s disease; PIAQ = Pfeffer Instrumental Activities Questionnaire; PSG = polysomnography; PSQI = Pittsburgh Sleep Quality Index; PVT = psychomotor vigilance task; rpm= revolutions per minute; SCI = spinal cord injury; SIS = Stroke Impact Scale.

**Table 2:** Modified NOS scale scores of the included studies.

Author, Year	Study Type	Selection	Treatment Protocol	Outcome(s)	Total (0 to 7)
Tidman, 2020 [52]	One-group pre-test and post-test	*	**	***	6/7
Fulk, 2020 [43]	Cross-sectional secondary analysis of longitudinal data	*	*	***	5/7
Colledge, 2017 [51]	Exploratory pre–post-test investigation with a 6-month follow-up	*	*	***	5/7
De Mello, 2002 [50]	No controlled trial		**	***	5/7
Nascimento, 2014 [49]	No controlled trial	**	*	***	6/7

* Each asterisk indicates an assigned point (** = 2 points; *** = 3 points).

## Data Availability

Not applicable.

## Appendix A

Search Strategy in MEDLINE (PubMed)

(((sleep disorder*) AND (rehabilitation)) AND (physical therapy modalities)

Search Strategy in PEDro

- Sleep disorder* AND physical therapy
- Sleep disorder* AND rehabilitation
- Sleep disorder* AND physiotherapy

Key Words

“sleep disorders”

“rehabilitation”

““physical therapy modalities”

“neurological diseases”
